# Coexistence field trials between MON810 and conventional maize in Mallorca as a basis for a regional regulatory proposal based on scientific evidence in the times of genome editing

**DOI:** 10.1007/s11248-024-00384-y

**Published:** 2024-05-07

**Authors:** Juan Antonio Vives-Vallés, Maria Corujo, Maria Pla, Jeroni Galmés

**Affiliations:** 1 Department of Private Law / Agro-Environmental and Water Economics Institute (INAGEA), University of the Balearic Islands, 07122 Palma, Illes Balears Spain; 2 08041 Madrid, Spain; 3 Institute of Food and Agricultural Technology, Universitat de Girona, 17004 Girona, Catalunya Spain; 4 Department of Biology / Agro-Environmental and Water Economics Institute (INAGEA), University of the Balearic Islands, 07122 Palma, Illes Balears Spain

**Keywords:** Biotechnology, Coexistence, EU law, GMOs, Mediterranean conditions, Sea breezes

## Abstract

This paper reports the first coexistence field trials between transgenic and conventional maize carried out under Mediterranean island conditions. Their purpose was to assess the local validity of pollen barriers and sowing delays as coexistence strategies as a basis for a regional regulation on the subject. Two field trials were performed in two agricultural states of Alcudia and Palma, in Mallorca (Spain). In the first one, two adjacent plots were synchronously sown with conventional and transgenic maize, respectively. In the second trial, the previous design was replicated, and two additional plots sown with GM maize were added, paired with their respective conventional recipient plots sown 2 and 4 weeks later. All conventional plots were located downwind from their respective GM plots. Of the two conventional plots in sowing synchrony, only one of them required a 2.25 m pollen barrier to meet the 0.9% labeling threshold. A 4-week sowing delay between GM and non-GM plots proved to be enough to keep the GM content of the recipient plots below the legal threshold. However, with a 2-week sowing delay additional coexistence measures such as pollen barriers might be needed, as suggested in the literature. Results are consistent with previous research conducted in the northeast of Spain, thus validating in the island’s agroclimatic conditions a model successfully tested in that peninsular region which allows to accurately estimate the need and width of pollen barriers. The results presented here could perhaps be extrapolated to other islands, coastal areas, and regions with stable prevailing winds during the maize flowering season.

**Supplementary Information:**

The online version contains supplementary material available at 10.1007/s11248-024-00384-y.

## Introduction

### Coexistence, back in fashion

After several years of virtually disappearing as a subject of study, coexistence is once again becoming a topic of interest throughout the EU. For some years now, with the emergence of New Genomic Techniques (NGTs) at the policy level in the EU, concerns focus mainly on “*coexistence with organic agriculture*” (European Commission 2021a, p. 28). Explicit references to “*coexistence*” are found in recital (38) of the NGT Proposal (European Commission, 2023b), which even devotes Art. 24 to the matter. But, as the European Commission acknowledges in the “*Explanatory Memorandum*” supporting the NGT Proposal, worries from the organic sector go far beyond the scope of the NGT Proposal itself (European Commission 2023a, p. 8). Thus, amendments to that proposal during its legal procedure ahead or additional legislative acts afterwards to further develop coexistence cannot be ruled out. Those amendments all the more likely considering: a) the difficulty of detection inherent to certain NGT uses (National Academies of Sciences Engineering and Medicine 2016; SAM 2018) which the NGT Proposal also reflects (European Commission, 2023b); b) the European Commission’s firm commitment to organic farming (European Commission, 2021b; Purnhagen et al. 2021); and, c) the special vulnerability of organic farming with regard to coexistence with GM crops (Demont and Devos 2008; Verrière 2015) which the NGT Proposal also echoes in relation to “*NGT plants*” (European Commission 2023a, p. 8).

### Coexistence, more likely in Spain and the Balearic Islands

Spain, the Balearic Islands and Mallorca exhibit some remarkable singularities relating to the likelihood of real coexistence situations. Spain holds 96% of the cultivated area of MON810 maize in the EU (Álvarez et al. 2022) and is the second EU member state in organic farming acreage, following France (European Commission 2023b).

The Balearic Islands are the fourth Spanish region in terms of utilised agricultural area registered in organic farming in relation to the overall utilised agricultural area of the region, with 16.4% based on 2021 data (GOIB 2022). In 2022, 119.47 hectares of maize MON810 were grown in the Balearic Islands (MAPA 2022a) out of 689 hectares of maize in the region, including rainfed, forage and sweet maize (MAPA 2022b), accounting for a 17% of the total Balearian maize acreage.

### Coexistence in the Balearic Islands, also a potentially sensitive societal issue

The Balearic Islands, and Mallorca, are also major tourist destinations (INE, IBESTAD). Most tourists come from Germany, Italy, the United Kingdom, France and the Netherlands (IBESTAD), countries which introduced national or regional bans to the cultivation of maize MON810 under Commission Implementing Decision (EU) 2016/321^1^ (Vives-Vallés 2016). The prohibition of the cultivation of transgenic maize and, in general, of GMOs, is a priority of the Majorcan Association of Organic Agriculture Producers, APAEMA (Terrasa 2016). The regional Parliament passed a law promoting organic farming and aiming at banning GMO cultivation in the Balearic Islands by invoking Directive (EU) 2015/412 (Vives-Vallés, 2021). The latter objective has not been achieved so far, but it reflects the sensitivity of the issue in the region from a socio-political perspective.

### The island’s agro-climatic conditions as distinctive coexistence factors

Gene flow through pollen dispersal is one of the main agroclimatic factors affecting coexistence between crops at the field level (Devos et al. 2013, p. 382). This factor is influenced by “*wind direction and speed*” (Devos et al. 2009, p. 20). The “*size and shape of the fields*” (Messeguer et al. 2006, p. 640) also has a significant influence on coexistence (Melé et al. 2014a, b, 2015; Messeguer et al. 2006).

The “*Technical and Best Practice Guidelines for the Cultivation of Bt Maize*” (translated) from the Spanish National Plant Breeders’ Association ANOVE suggests either to keep “*a strip of 12 rows of conventional maize of similar cycle*” separating neighboring fields, or a temporal isolation of sowings “*of 4 weeks in April, or two weeks in May*” to meet the EU labelling threshold (translated from ANOVE 2021, p. 1). These suggestions were subject to (positive) “*validation* […] *in the ‘worst- case scenario’*” by Nadal et al. (2016, p. 24), both empirically at a field level in Catalonia, and “in silico” with the GIMI 2 tool built by Melé et al. (2014a, b).

However, according to APAEMA, the long reach of GM maize “*pollen*”, along with Majorcan agrarian structure based on “*small plots* […] *makes coexistence between transgenic crops and organic crops impossible: if the seeds are contaminated, they can no longer be used in organic farming*” (translated from APAEMA, in Terrasa 2016).

The “*sea breeze*” phenomenon, long studied by the specialized literature (Fisher 1960; Rotunno 1983), is believed to significantly condition “[t]*he climate and the atmospheric environment in Mediterranean coastal regions*”, and “*is* [even] *more pronounced in the islands*” (Melas et al. 2000, p. 516). Such phenomenon is also known to alter pollen fluxes, levels, and reach (Aguilera and Ruiz Valenzuela 2009; Alarcón et al. 2022; Gassmann et al. 2002; Gassmann and Pérez 2006; Greene et al. 2008; Negral et al. 2021; Raynor et al. 1974; Smith and Emberlin 2006; Viner et al. 2017; Williams 2020).

The bays of Palma and Alcúdia are the areas where the sea breeze phenomenon is of greatest significance in Mallorca (Alomar-Garau and Grimalt-Gelabert 2022; Alomar Garau 2013; Ponce de León and Orfila 2013; Ramis et al. 1990; Ramis and Romero 1995; Ramis Noguera 1998; Romero and Ramis 1996), with a prevalence of up to “*80% of the days in July and 76% in August*” (Ramis and Romero 1995, p. 5). The most important maize growing areas in the Balearic Islands are close to those bays (CAIB 2012, 2013), and July and August are very relevant in terms of maize pollination in Northeastern Spain (Melé et al. 2014b; Nadal et al. 2016) and the Balearic Islands.

Thus, under these socio-political, farming, and agroclimatic constraints, robust but proportionate coexistence rules are essential to successfully balance all the interests at stake. Science has proven to be an invaluable tool in this endeavor (Devos et al. 2009). This paper reports the results of the first coexistence field trials conducted in Mallorca under the insular farming and agroclimatic conditions summarized above. Their purpose was to evaluate pollen barriers and delayed sowings as coexistence strategies under such conditions as a scientific basis for a regional regulatory proposal on coexistence. Although in the field trials transgenic maize was used, the results obtained could be eventually extrapolated to coexistence situations involving gene-edited maize via the use of NGTs.

## Material and methods

### Field trials general design

Two field trials were conducted in two agricultural estates close to the bays of Alcúdia at the NW of Mallorca in 2013 (Trial 1), and Palma at the SW of Mallorca in 2015 (Trial 2), to evaluate the effect of pollen barriers (Trials 1 and 2) and of sowing asynchrony (Trial 2).

**Fig. 1:**
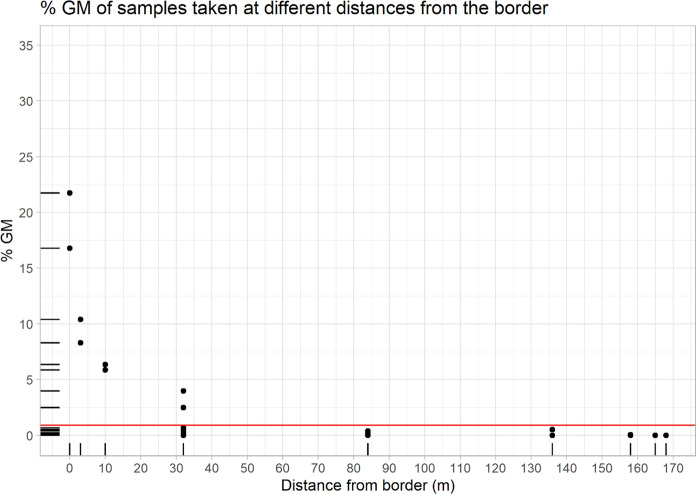
MON810 content of samples collected at the different sampling points of the recipient plot of Trial 1. On the abscissa axis: distance from the closest border to the GM plot; on the ordinate axis: the MON810 content of each of the samples, with the 0.9% threshold line in red. Figure plotted with R

In Trial 1, pollen gene flow from the GM donor plot to the conventional recipient plot was forced by placing the plots adjacent to each other (Fig. 1S), with the conventional recipient plot downwind the GM donor plot, and with the slope factor also in favour of such cross-fertilization. Pioneer seeds from GM PR34A27 and conventional P1114 maize varieties (500 FAO cycle) were sown, with a sowing density of 88,000 seeds/ha, a sowing pattern of 0.15 m between plants × 0.75 m between rows, and a sowing depth of 0.04–0.06 m, both the GM donor plot and the GM recipient plot sown on May 18th. The rows run parallel to the length of the field, separated by parallel sprinkler rows spaced 15 m apart. The usual cultivation practices in Mallorca were followed, involving irrigation, fertilization, and insecticide treatments.

**Fig. 2:**
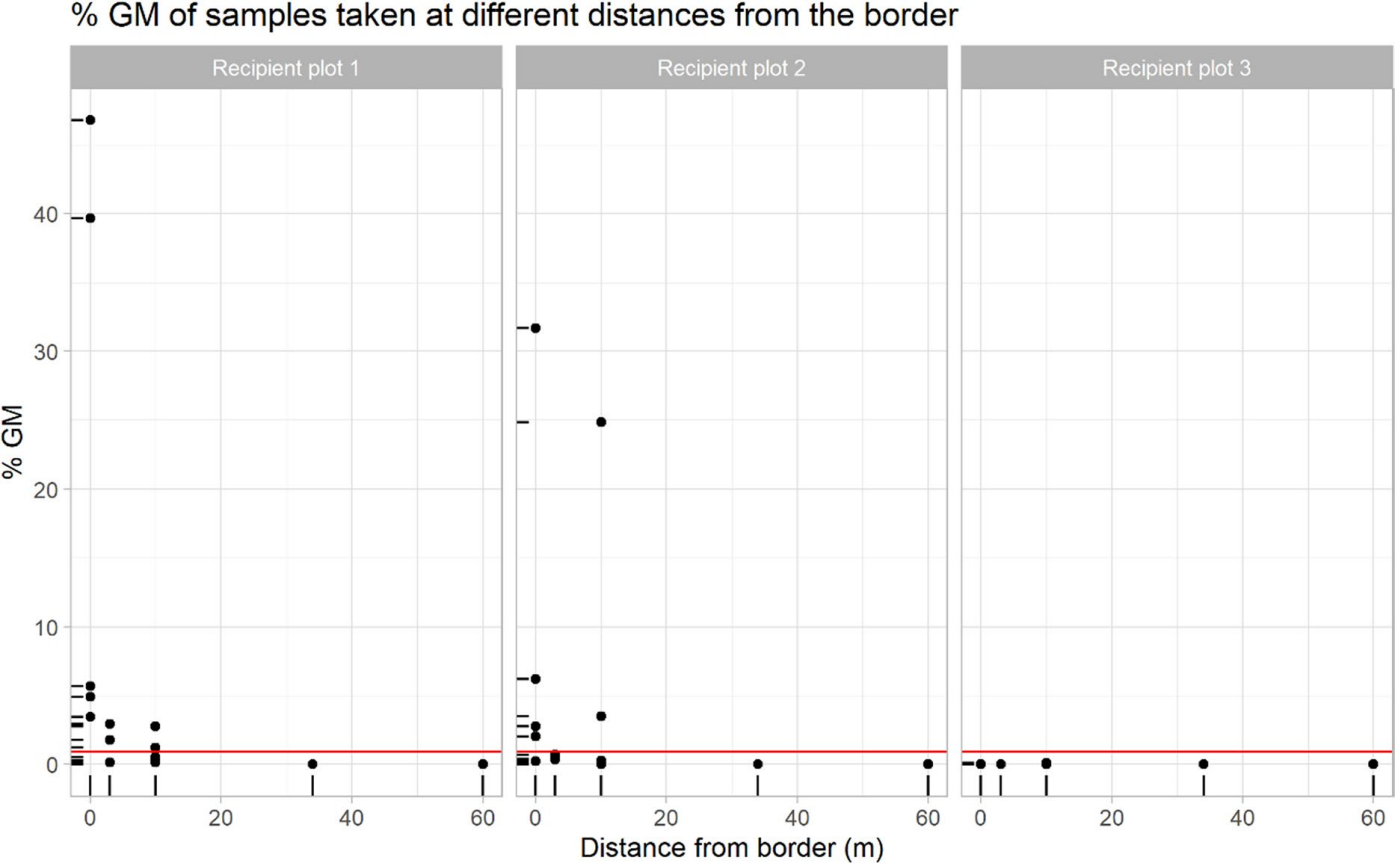
MON810 content of samples collected at the different sampling points of the recipient plots of Trial 2. From left to right, the following graphs are shown: 1) recipient plot 1 (sowing synchrony), 2) recipient plot 2 (15 days sowing delay), and 3) recipient plot 3 (29 days sowing delay). In each graph, on the abscissa axis: distance from the closest border to the homologous GM plot; on the ordinate axis: the MON810 content of each of the samples, with the 0.9% threshold line in red. Figure plotted with R. Only samples up to 60 m from that border are shown (all other samples further away had MON810 values below the detection level). (Color figure online)

Trial 2 was carried out in a different year and in a different area, under relatively dissimilar conditions. In this case, the design of Trial 1 shown in Fig. 1S was essentially tripled (although without relevant slope differences between homologous GM and non-GM plots), isolating each of the replications (comprising a GM donor plot paired with a conventional recipient plot) by a 30 m wide buffer zone sown with conventional maize (Fig. 2S). Pioneer seeds from GM PR33Y72 and conventional PR33Y74 maize varieties (600 FAO cycle) were sown, with a density of 88,000 seeds/ha for conventional plots and buffer zones, and of 80,000 seeds/ha for GM plots; and the same sowing pattern, sowing depth, and orientation of the maize and sprinkler rows in relation to the length of the plots as in Trial 1. Conventional recipient plot 1, conventional buffer zones, plus all GM donor plots were sown on June 2nd, while conventional recipient plots 2 and 3 were sown respectively on June 17th and July 1st (that is, circa. 2- and 4-weeks sowing delay, respectively, with GM donor plots 2 and 3). As in Trial 1, the fields were irrigated, fertilized, and insecticide treatments were applied according to usual practices in the island.

### Meteorological, flowering and pollen dispersal estimates

Meteorological data were collected from two stations of the Spanish State Meteorological Agency AEMET: a) for Trial 1, the “*AEMET SA POBLA SA CANOVA*” with code B691Y, located circa. 12 km distance from the field of that trial; b) for Trial 2, the “*AEMET PALMA DE MALLORCA/SON SAN JUAN*” with code B278, located only 6 km from the field of that trial.

Flowering peaks were determined by direct visual observations in the field. Flowering periods were fixed on the basis of the observed flowering peaks and the observations from the relevant literature which sets pollen flowering intervals between 5 and 8 days (Della Porta et al. 2008, p. 261; Halsey et al. 2005, p. 2173; Messeguer et al. 2006, p. 640; Wolt et al. 2003, p. 240) and flowering pics around 2–3 days after the flowering start (Wolt et al. 2003, p. 240). Pollen dispersal hours were assumed on the basis of the relevant literature, the daily dispersal time frame between 06:00 a.m. and 06:00 p.m. (Halsey et al. 2005; Langhof et al. 2008) and the maximum dispersal hours between 09:00 a.m. and 02:00 p.m. (Della Porta et al. 2008; Halsey et al. 2005).

### Sampling

**Fig. 3:**
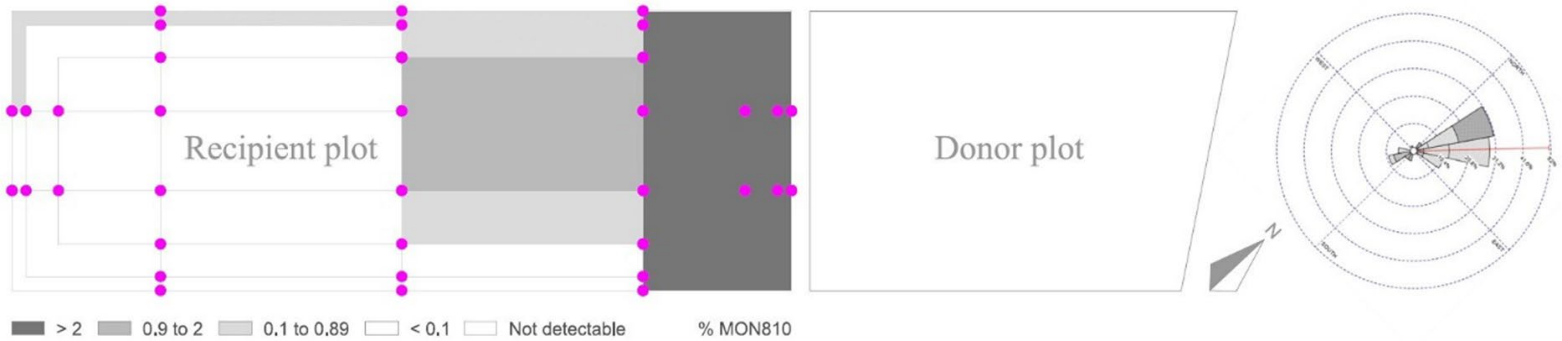
MON810 contents per quadrant in the conventional recipient plot of Trial 1 (sowing synchrony), calculated as the average of the MON810 content of the samples collected in the points at the corners of each quadrant, following Messeguer et al. (2006). A wind rose diagram representing wind direction and speed (m/s) between 06:00 a.m. and 06:00 p.m. during the flowering period has been added on the right

**Fig. 4:**
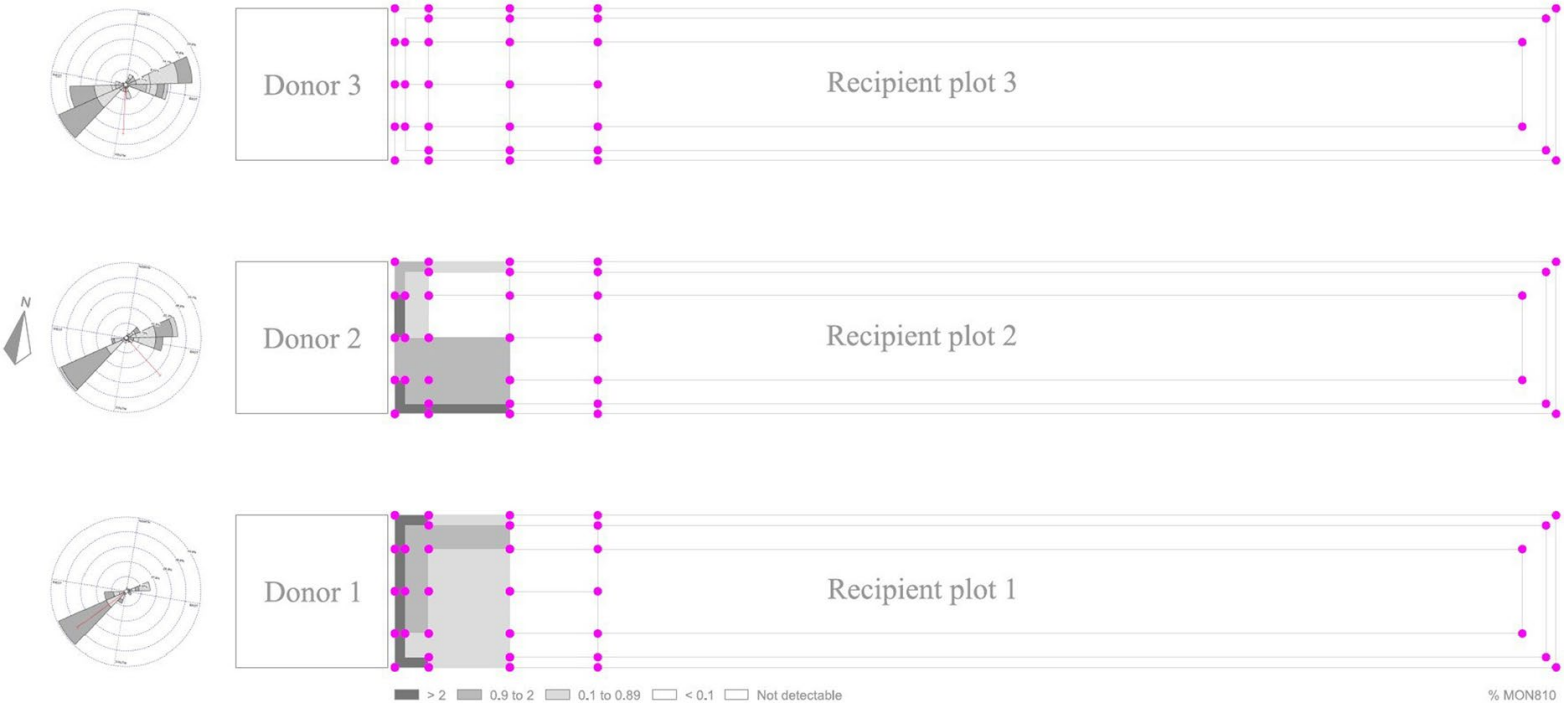
MON810 contents per quadrant in conventional recipient plots 1 (sowing synchrony), 2 (15 days sowing delay), and 3 (29 days sowing delay) of Trial 2, calculated as the average of the MON810 content of the samples collected in the points at the corners of each quadrant, following Messeguer et al. (2006). Wind rose diagrams representing wind direction and speed (m/s) between 06:00 a.m. and 06:00 p.m. during the flowering periods of conventional recipient plots have been added on the left of each donor plot

The Messeguer et al. (2006) stratified sampling methodology was used in both trials, adapted to the geometry of the conventional recipient plots. Sampling points locations at the conventional recipient plots are shown in Fig. 3S and 4S. In Trial 2, the same sampling scheme was followed in the three recipient plots. At each of the sampling points a sample was collected, consisting of three cobs randomly collected within a 1 m radius from that point. Some samples were also collected in the donor plots, and 1 kg of sowing seeds were kept for purity control. Where relevant, some leaf DNA tests were also performed to determine the origin of the transgene (gene flow or seed).

### DNA extraction and qPCR

From each of the samples, the cobs were manually shelled, the kernels mixed and grounded using a GRINDOMIX GM 200 blade mill (Retsch GmbH, Haan, Germany). 200–250 mg of the resulting powder was used for the DNA extraction with the NucleoSpin^®^ Food kit (Macherey–Nagel GmBH, Düren, Germany), conducting one measurement per sample, as described in Corujo (2016).

DNA quantification was performed using a spectrophotometer (NanoDrop, Wilmington, USA), reading the D.O. at 260 nm. The qPCRs were carried out using a Light Cycle 480 real-time PCR Instrument (Roche Holding AG, Basel, Switzerland), fluorescence monitored with Light Cycle 480 software v 1.5.0 (Roche Holding AG, Basel, Switzerland). Detection and quantification of the MON810 event was conducted by the validated JCR method by Shindo et al. (2002), starting from 100 µg DNA, and using Premix Taq™ DNA Polymerase (TaKaRa Taq™ Version 2.0) (Takara Bio Inc., Shiga, Japan).

### Determination of the gene flow distribution and the GM content of the fields

The gene flow distribution and the GM content per quadrant was estimated at the experimental level by the Messeguer et al. (2006) methodological approach.

The overall GM content of each plot was reached through the application of three approaches: (i) on the basis of GM content at every quadrant as described in Messeguer et al. (2006); (ii) with an *ex-post* adaptation of the experimental approach described in Melé et al. (2015); and, (iii) in silico through the use of the GIMI 2 tool built by Melé et al. (2014a, b). Melé et al. (2015) allows to estimate both the “*external*” MON810 content (i.e., unrelated to the field’s own impurities) and the “*overall*” MON810 content (i.e., also considering potential internal impurities); while the GIMI 2 tool by Melé et al. (2014a, b) allows to predict the GM content of a certain plot on the basis of a given agricultural structure (i.e., including the size and shape of the agricultural plots, as well as their arrangement in space, their GM or non-GM “*type*”, and their “*flowering dates*”).

#### ***Adaptation of ***Melé et al. (2015)*** for the estimation of the overall GM content***

The overall GM content of the recipient plots was estimated through the application of equations “*(4)*” and “*(2)*” from Melé et al. (2015) adapted to the particular sampling designs of the trials. Particularly, the results considered were those from the samples collected at the sampling points located 3 m distance from the borders for both trials 1 and 2 (red dots from Figs. 3S and 4S, respectively), plus those from the samples collected at the inner perimeter for Trial 1 (cyan dots from Fig. 3S), and from the samples at the corners of the two central quadrants at 34 m and 60 m of the GM donor plot for Trial 2 (cyan dots from Fig. 4S).

#### ***Application of ***Melé et al. (2015)*** for the estimation of the border/perimeter rows***

Additionally, the *ex-post* application of Melé et al. (2015) allowed to estimate the needed width of a border or perimeter sown with conventional maize to keep the GM content of conventional recipient plots below the labelling threshold (pollen barriers). Formulas “*(11a)*”, “*(11b)*”, and “*(9b)*” from Melé et al. (2015) were used, by considering the mean of the GM content in the samples collected at 3 m distance from the borders of each plot.

## Results

Figure 1 portrays the MON810 content of each sample collected at each sampling point on the conventional recipient plot of Trial 1. No samples with an estimated MON810 content higher than 0.9% were found farther than 32 m from the GM donor plot (Fig. 1 and Table 1S). Only 2 out of 8 samples collected at 32 m from the GM donor plot yielded GM contents higher than 0.9% (Fig. 1 and Table 1S). MON810 contents in samples collected at the conventional recipient plots of Trial 2 are shown in Fig. 2. In Trial 2, MON810 contents above the 0.9% threshold were detected up to 10 m from the donor field in plots 1 (sowing synchrony) and 2 (15 days sowing delay) (Fig. 2, and Table 2S). In plot 3 (29 days sowing delay), only 3 samples showed detectable MON810 levels, all three below 0.1% (and within 10 m from the GM border).

Figures 3 and 4 show the MON810 contents per quadrant of the conventional recipient plots from Trials 1 and 2 estimated by applying the procedures described in Messeguer et al. (2006). In Trial 1, all quadrants up to 32 m far from the GM donor plot exceeded 2% MON810, and 2 out of 7 quadrants from 32 to 84 m distance from the GM plot had between 0.9 and 2% MON810 (Fig. 3). In plot 1 of Trial 2 (sowing synchrony), all quadrants bordering the donor field exceeded 2%, while 3 quadrants located between the 3 and 10 m lines, and 1 quadrant between the 10 and 34 m lines yielded MON810 values in the 0.9–2% range (Fig. 3). There was one more quadrant exceeding 0.9% MON810 in recipient plot 2 (15 days sowing delay) than in recipient plot 1 of the same trial (Fig. 3). However, the overall GM content was higher in plot 1 than in plot 2 (Table 1). With 29 days sowing delay (plot 3), the MON810 content was below the limit of detection or negligible (below 0.1%) in all quadrants (Fig. 3).

**Table 1:** MON810 content (%) of the conventional recipient plots of Trials 1 and 2, estimated by different experimental (Messeguer et al. (2006), Melé et al. (2015)) and in silico (GIMI 2 tool by Melé et al. (2014a, b)) methods

Parameters/results	Trial 1 Recipient plot	Trial 2		
		Recipient plot 1	Recipient plot 2	Recipient plot 3
MON810 content (%) estimated by:				
Messeguer et al. 2006:	1.222	0.162	0.124	0.001
Melé et al. (2015):				
“*Overall*” MON810 content (%):	1.475	0.306	0.089	0.008
“*External*” MON810 content (%):	1.200	0.386	0.113	0.010
GIMI 2 (Melé et al. 2014a, b):	0.27	0.22	0.02	0.00
Perimetral border (Melé et al. 2015):	2.25	0.00	0.00	0.00

The Melé et al. (2015) method allows to distinguish the “*external*” MON810 content from the “*overall*” MON810 content (which also takes into account the field’s own impurities). The perimetral border is the width (m) of conventional maize required to keep the MON810 contents of recipient conventional plots below the labelling threshold

These values, along with the quadrant surfaces, allowed estimating the overall MON810 contents in the whole recipient field using the procedures described in Messeguer et al. (2006) (Table 1). Only the conventional recipient plot of Trial 1 exceeded 0.9% MON810, with 1.222% (Table 1). GM content values for all conventional recipient plots of Trials 1 and 2, estimated by Messeguer et al. (2006), Melé et al. (2015), and in silico, using the GIMI 2 tool by Melé et al. (2014a, b), are shown in Table 1. The perimetral borders sown with conventional maize that would be needed to keep the MON810 content of the recipient plots below 0.9% are also shown in Table 1 and were estimated using the Melé et al. (2015) approach. None of the conventional plots in Trial 2 required a perimetral border.

## Discussion

Estimates of GM contents with the different methodologies and predictive models are consistent (Table 1). The application of GIMI 2 by Melé et al. (2014a, b) to Trial 1 deviates slightly from the experimental approaches (Table 1). Such a deviation could be due to the especially forced conditions of that trial, with a pronounced effect of sea breezes during the flowering period (Figs. 1S, 9S), and with the slope also in favor of gene flow. “*[W]ind is the major factor for maize cross-pollination*” (Messéan et al. 2006, p. 27) and GIMI 2 was not designed under conditions as severe as those of the Mallorca trials. Indeed, Messeguer et al. (2006, p. 640) found that: “*In our study, we detected some wind effects, mainly elongating the flow along the edges of the fields, but any overall effect was diluted because the donor fields faced different directions.*” Trials under Messeguer et al. (2006) were conducted in the same area on which the GIMI 2 model was built, under (unforced) field conditions, and over very large areas with many alternate GM and conventional fields, offsetting the wind effect.

The study conducted shows that coexistence between MON810 maize and non-GM maize is feasible under typical agricultural conditions in Mallorca. Moreover, given the high prevalence of the sea breeze phenomenon, the need for pollen barriers or sowing delays could eventually be reduced if care is taken to place the MG fields upwind of conventional and organic fields. Wind unpredictability has led experts to claim that “*this parameter could not be used for developing proposals for coexistence measures*” (Czarnak-Klos and Rodríguez-Cerezo 2010, p. 37). Prevalence or stability, however high, does not mean absolute certainty. But the strong prevalence of the sea breezes phenomenon in Trials 1 and 2 areas, both reported by the literature (Ramis and Romero 1995) and prevailing also in the context of the trials from June to August (Figs. 5S, 6S, 7S, 9S, 10S, 11S), suggests at least the need to further explore the potential role of this phenomenon in the design of coexistence strategies in Mallorca, and, eventually, in other insular, coastal areas, or in other regions with a strong prevalence of dominant winds during pollination. As a result of these new modeling and design efforts, more adjusted and proportionate coexistence measures might be eventually developed.

Four weeks sowing delay were sufficient to keep the GM content of the recipient fields below the labelling threshold, but it could not be concluded that 2 weeks sowing delay would be sufficient in the case of extra late sowings. Subsequent DNA tests carried out on suspicious leaf samples showed some positive results, which could fit under the pattern of the “*hot-spot*” phenomenon described in the literature (e.g., Bannert 2006). This might explain the GM content results in the 2 weeks sowing delay scenario. In any case, implementing additional coexistence strategies (as suggested, e.g., by Czarnak-Klos and Rodríguez-Cerezo 2010) such as pollen barriers would easily make coexistence feasible, also under a 2 weeks sowing delay.

It must be noted, however, that delayed sowings may affect yields, particularly in regions further north than the Mediterranean (Messeguer et al. 2006; Devos et al. 2009), reducing the possibility of extrapolating these measures to “*non-Mediterranean regions where the window of suitable weather conditions is too short to postpone sowing*” (Devos et al. 2009, p. 19). Although the field trials under this study were not designed to assess the impact of delayed sowings on yield, a decline in yield was reported, which was particularly pronounced in the later sown plots from Trial 2. It should be noted that the window for sowing maize in Mallorca expands at least one month before the trials reported here were initiated, thus leaving room for the application of sowing delays under normal Majorcan farming conditions.

## Conclusion

In the field trials conducted in Mallorca, pollen barriers were required in only one plot, in which a perimetral border of just 2.25 m would have sufficed to control cross-pollination. Under the experimental conditions of the trials here reported ‒defined by the prevalence of sea breezes during the flowering time (conventional recipient plots located downwind), late to extra-late sowings, and a smallholding farming setting‒ 4 weeks sowing delay between GM and non-GM fields proved to be enough to comply with the labeling threshold. However, the study does not allow to conclude that 2 weeks sowing delay in that context would be sufficient in all cases to keep the GM content at bay. But even in that scenario, effective coexistence could be easily achieved by resorting to alternative or additional coexistence measures, such as pollen barriers.

Field trials between MON810 GM maize and conventional maize conducted in Mallorca show that coexistence on the island is feasible. Moreover, previous methodologies and models such as those of Messeguer et al. (2006) and Melé et al. (2015), designed and successfully tested in the Iberian Peninsula, have proven to work well also in Majorcan insular conditions.

Smallholdings, worrying APAEMA (Terrasa 2016), and the sea breeze phenomenon, widely reported by the specialized literature and prevailing also during the trials, appear to make coexistence a bit harder in the island. However, the effect of a reduced field size on coexistence is already considered by models such as Melé et al. (2015). As for the sea breeze phenomenon on the island, given its stability, it could be more an advantage than a drawback, since, at the very least, it could provide a basis for recommending conventional fields to be placed upwind of GM fields, thus reducing the impact of cross-pollination. This factor could even be considered in the design of more proportionate coexistence strategies.

The results and nuances of this study could perhaps be extrapolated to other islands and coastal areas, allowing better and more “*proportionate*” solutions (in line with: Demont and Devos 2008; Devos et al. 2009) for these regions, where constraints such as the availability of arable land, the scarcity of resources, etc., or concerns such as food security and climate change, probably pose a much greater challenge to effective coexistence than in continental areas that can rely on the *arrière-pays* and on road and rail communications. The potential success of the European Commission’s NGT Proposal, and the explicit recognition of the need for additional “*coexistence measures*” it contains, now in relation to certain “*NGT plants*”, further reinforces the potential interest of the results of this study, and, in general, the need to enable coexistence with science-based and proportionate approaches.

### Supplementary Information

Below is the link to the electronic supplementary material.

Supplementary file1 (DOCX 866 KB)

## Footnotes

1 Commission Implementing Decision (EU) 2016/321 of 3 March 2016 adjusting the geographical scope of the authorisation for cultivation of genetically modified maize (*Zea mays* L.) MON 810 (MON-ØØ81Ø-6) [2016] OJ L 60/90.

## References

[CR1] Aguilera F, Ruiz Valenzuela L (2009). Study of the floral phenology of *Olea europaea* L. in Jaén province (SE Spain) and its relation with pollen emission. Aerobiologia (bologna).

[CR2] Alarcón M, Periago C, Pino D (2022). Potential contribution of distant sources to airborne Betula pollen levels in Northeastern Iberian Peninsula. Sci Total Environ.

[CR3] Alomar Garau G (2013) Las brisas marinas y su significación geográfica. El caso de Mallorca. Sémata Ciencias Sociais e Humanidades 25:7–28. https://revistas.usc.gal/index.php/semata/article/view/1152/1652. Accessed 4 May 2024

[CR4] Alomar-Garau G, Grimalt-Gelabert M (2022). Impacts of coastal breezes on the environment and human life: the case of Mallorca (Western Mediterranean). Coasts.

[CR5] Álvarez F, Martín Camargo A, Messéan A (2022). Assessment of the 2020 post-market environmental monitoring report on the cultivation of genetically modified maize MON 810 in the EU. EFSA J.

[CR6] ANOVE (2021) Guía Técnica y de Buenas Prácticas para el Cultivo de maíz Bt. ANOVE, Madrid. https://www.anove.es/wp-content/uploads/2021/08/Guia-2021-cultivo-Maiz-Bt.pdf. Accessed 23 Jun 2023

[CR7] Bannert M (2006) Simulation of transgenic pollen dispersal by use of different grain colour maize. Swiss Federal Institute of Technology Zurich. http://www.ask-force.org/web/Coexistence/Bannert-ThesisETHZ-Maize-2006.pdf. Accessed 24 Dec 2015

[CR8] CAIB (2012) Datos obtenidos procedentes del Registre de superfícies agrícoles cultivades amb organismes modificats genèticament (OMG) i d’alliberament voluntari i utilització confinada d’OMG: 2012

[CR9] CAIB (2013) Datos obtenidos procedentes del Registre de superfícies agrícoles cultivades amb organismes modificats genèticament (OMG) i d’alliberament voluntari i utilització confinada d’OMG: 2013

[CR14] Corujo Besga M (2016) Tecnologías ómicas para la evaluación de riesgos de las plantas modificadas genéticamente. Universitat Autònoma de Barcelona (UAB). https://tdx.cat/handle/10803/394064. Accessed 3 May 2024

[CR15] Czarnak-Klos M, Rodríguez-Cerezo E (2010) Best practice documents for coexistence of genetically modified crops with conventional and organic farming: 1 Maize Crop Production. Publications Office of the European Union, Luxemburgo. https://op.europa.eu/en/publication-detail/-/publication/d9d74de7-e1cd-429c-a56d-0a4f62052d45/language-en. Accessed 16 Jul 2023

[CR16] Della Porta G, Ederle D, Bucchini L (2008). Maize pollen mediated gene flow in the Po valley (Italy): source-recipient distance and effect of flowering time. Eur J Agron.

[CR17] Demont M, Devos Y (2008). Regulating coexistence of GM and non-GM crops without jeopardizing economic incentives. Trends Biotechnol.

[CR18] Devos Y, Demont M, Dillen K (2009). Coexistence of genetically modified (GM) and non-GM crops in the European Union. A Review Agron Sustain Dev.

[CR19] Devos Y, Dillen K, Demont M (2013). How can flexibility be integrated into coexistence regulations? A review. J Sci Food Agric.

[CR10] European Commission (2021a) Commission staff working document[:] Study on the status of new genomic techniques under Union law and in light of the Court of Justice ruling in Case C-528/16[.] Brussels, 29.4.2021[.] SWD(2021) 92 final. Brussels, Belgium. https://food.ec.europa.eu/system/files/2021-04/gmo_mod-bio_ngt_eu-study.pdf. Accessed 9 Oct 2021

[CR11] European Commission (2021b) European Green Deal: Commission presents actions to boost organic production. In: Press release. https://ec.europa.eu/commission/presscorner/detail/en/IP_21_1275. Accessed 14 May 2023

[CR12] European Commission (2023a) Proposal for a Regulation of the European Parliament and of the Council on plants obtained by certain new genomic techniques and their food and feed, and amending Regulation (EU) 2017/625 (COM(2023) 411 final). 70. https://food.ec.europa.eu/document/download/c03805a6-4dcc-42ce-959c-e4d609010fa3_en?filename=gmo_biotech_ngt_proposal.pdf. Accessed 5 Aug 2023

[CR13] European Commission (2023b) Agricultural Market Brief Organic[.] Organic farming in the EU[.] A decade of organic growth[.] January 2023. https://agriculture.ec.europa.eu/document/download/df01a3c7-c0fb-48f1-8eca-ce452ea4b8c2_en?filename=agri-market-brief-20-organic-farming-eu_en.pdf. Accessed 16 Jul 2023

[CR20] Fisher EL (1960). An observational study of the sea breeze. J Meteorol.

[CR21] Gassmann MI, Pérez CF, Gardiol JM (2002). Sea-land breeze in a coastal city and its effect on pollen transport. Int J Biometeorol.

[CR22] Gassmann MI, Pérez CF (2006). Trajectories associated to regional and extra-regional pollen transport in the southeast of Buenos Aires province, Mar del Plata (Argentina). Int J Biometeorol.

[CR23] GOIB (2022) Agricultura elaborará un plan estratégico de la producción agraria ecológica para lograr el 25 % de la SAU en 2030. https://www.caib.es/pidip2front/jsp/es/ficha-convocatoria/strongagricultura-strongstrongelaboraraacute-un-plan-estrateacutegico-de-la-produccioacuten-agraria-ecoloacutegica-para-lograr-el-25-de-la-sau-ennbsp2030strong. Accessed 19 Jul 2023

[CR24] Greene DF, Quesada M, Calogeropoulos C (2008). Dispersal of seeds by the tropical sea breeze. Ecology.

[CR25] Halsey ME, Remund KM, Davis CA (2005). Isolation of maize from pollen-mediated gene flow by time and distance. Crop Sci.

[CR26] IBESTAT. https://ibestat.caib.es/ibestat/inici. Accessed 18 Jun 2023

[CR27] INE. https://www.ine.es. Accessed 18 Jun 2023

[CR28] Langhof M, Hommel B, Hüsken A (2008). Coexistence in maize: do nonmaize buffer zones reduce gene flow between maize fields?. Crop Sci.

[CR29] MAPA (2022a) Estimación de la superficie total de variedades OMG cultivadas en España. https://www.mapa.gob.es/es/agricultura/temas/biotecnologia/estimacionsuperficietotalomgespana2022_tcm30-631740.pdf. Accessed 19 Jun 2023

[CR30] MAPA (2022b) Encuesta sobre Superficies y Rendimientos de Cultivos. Resultados definitivos 2022. In: ESYRCE. https://www.mapa.gob.es/es/estadistica/temas/estadisticas-agrarias/boletin2022_tcm30-667379.pdf. Accessed 4 May 2024

[CR31] Melas D, Lavagnini A, Sempreviva A-M (2000). An Investigation of the boundary layer dynamics of Sardinia island under sea-breeze conditions. J Appl Meteorol.

[CR32] Melé E, Nadal A, Melé-Messeguer M, et al (2014a) GIMI 2.0 Web

[CR33] Melé E, Nadal A, Melé-Messeguer M, et al (2014b) GIMI 2: A Tool for Fast Estimation and Prediction of GMO Maize. AgBioForum 17:172–182. https://agbioforum.org/wp-content/uploads/2021/02/AgBioForum-17-3-172.pdf. Accessed 4 May 2024

[CR34] Melé E, Nadal A, Messeguer J (2015). Modeling gene flow distribution within conventional fields and development of a simplified sampling method to quantify adventitious GM contents in maize. Sci Rep.

[CR35] Messéan A, Angevin F, Gómez-Barbero M (2006) Technical Report EUR 22102 EN: New case studies on the coexistence of GM and non-GM crops in European agriculture. Sevilla. https://op.europa.eu/en/publication-detail/-/publication/cc3b24fa-f633-4b3a-8da0-0ee02d2d5199. Accessed 4 May 2024

[CR36] Messeguer J, Peñas G, Ballester J (2006). Pollen-mediated gene flow in maize in real situations of coexistence. Plant Biotechnol J.

[CR37] Nadal A, Pla M, Messeguer J (2016). Asynchronous flowering or buffer zones: technical solutions for small-scale farming. EuroChoices.

[CR38] National Academies of Sciences Engineering and Medicine (2016) Genetically engineered crops: experiences and prospects. The National Academies Press, Washington. https://www.ncbi.nlm.nih.gov/books/NBK424543/. Accessed 4 May 2024 28230933

[CR39] Negral L, Moreno-Grau S, Galera MD (2021). The effects of continentality, marine nature and the recirculation of air masses on pollen concentration: Olea in a Mediterranean coastal enclave. Sci Total Environ.

[CR40] Ponce de León S, Orfila A (2013). Numerical study of the marine breeze around Mallorca Island. Appl Ocean Res.

[CR41] Purnhagen KP, Clemens S, Eriksson D (2021). Europe’s farm to fork strategy and its commitment to biotechnology and organic farming: conflicting or complementary goals?. Trends Plant Sci.

[CR42] Ramis C, Romero R (1995). A first numerical simulation of the development and structure of the sea breeze on the Island of Mallorca. Ann Geophys.

[CR43] Ramis C, Jansà A, Alonso S (1990). Sea breeze in Mallorca. A Numerical Study Meteorol Atmos Phys.

[CR44] Ramis Noguera C (1998) L’embat a l’illa de Mallorca. Territoris 1:253–274. https://www.raco.cat/index.php/Territoris/article/download/116755/147663. Accessed 8 Oct 2021

[CR45] Raynor GS, Hayes JV, Ogden EC (1974). Mesoscale transport and dispersion of airborne pollens. J Appl Meteorol.

[CR46] Romero R, Ramis C (1996) A numerical study of the transport and diffusion of coastal pollutants during the breeze cycle in the Island of Mallorca. http://meteorologia.uib.eu/ROMU/formal/contam/contam.pdf. Accessed 21 Jun 2023

[CR47] Rotunno R (1983). On the linear theory of the land and sea breeze. J Atmos Sci.

[CR48] SAM (2018) Statement by the Group of Chief Scientific Advisors: A Scientific Perspective on the Regulatory Status of Products Derived from Gene Editing and the Implications for the GMO Directive. Brussels. https://op.europa.eu/en/publication-detail/-/publication/a9100d3c-4930-11e9-a8ed-01aa75ed71a1/language-en/format-PDF/source-94584603. Accessed 2 Dec 2019

[CR49] Shindo Y, Kuribara H, Matsuoka T (2002). Validation of real-time PCR analyses for line-specific quantitation of genetically modified maize and soybean using new reference molecules. J AOAC Int.

[CR50] Smith M, Emberlin J (2006). A 30-day-ahead forecast model for grass pollen in north London, United Kingdom. Int J Biometeorol.

[CR51] Terrasa M (2016) El conreu de transgènics guanya territori. In: Arabalears. http://www.arabalears.cat/balears/conreu-transgenics-guanya-territori_0_1699030227.html. Accessed 18 Nov 2020

[CR52] Verrière P (2015) Preventing GMO contamination. An overview of national “coexistence” measures in the EU. Bruselas. http://www.ifoam-eu.org/sites/default/files/ifoameu_policy_gmos_dossier_201412.pdf. Accessed 30 Aug 2016

[CR53] Viner BJ, Arritt RW, Westgate ME (2017). Examination of climatological wind patterns and simulated pollen dispersion in a complex island environment. Int J Biometeorol.

[CR54] Vives-Vallés JA (2016). Derecho de Cultivos Transgénicos: El conflicto entre el Derecho español y comunitario y el derecho a la libertad de empresa, a la luz de la nueva normativa opt-out.

[CR55] Vives-vallés JA (2021). Análisis de los últimos desarrollos legislativos a nivel de la CAIB en materia de cultivos modificados genéticamente: evolución, situación actual e implicaciones legales y empresariales. Rev Derecho La Competencia y La Distrib.

[CR56] Williams CG (2020). Atmospheric layering during peak pine pollen season. Grana.

[CR57] Wolt J, Peterson R, Bystrak P, Meade T (2003). A screening level approach for Nontarget insect risk assessment: transgenic Bt corn pollen and the monarch butterfly (*Lepidoptera: Danaidae*). Environ Entomol.

