# Supplementary material for: Coexistence field trials between MON810 and conventional maize in Mallorca as a basis for a regional regulatory proposal based on scientific evidence in the times of genome editing
Source: Transgenic Res. 2024 May 7;33(3):119–30. doi: 10.1007/s11248-024-00384-y (PMC11176244; doi:10.1007/s11248-024-00384-y)
Supplement: Supplementary file 1 — Supplementary file1 (DOCX 866 KB) [file 11248_2024_384_MOESM1_ESM.docx]

**Supplementary Material**

**Journal: Transgenic Research**

**Title: Coexistence field trials between MON810 and conventional maize in Mallorca as a basis for a regional regulatory proposal based on scientific evidence in the times of genome editing**

Authors: Juan Antonio Vives-Vallés^1*^, Maria Corujo, Maria Pla, Jeroni Galmés

^1^ University of the Balearic Islands, Agro-Environmental and Water Economics Institute / Dept. of Private Law, Palma 07122, Illes Balears, Spain, <https://orcid.org/0000-0001-7652-1599>

^*^ E-mail address: [juanantonio.vives@uib.eu](mailto:juanantonio.vives@uib.eu).


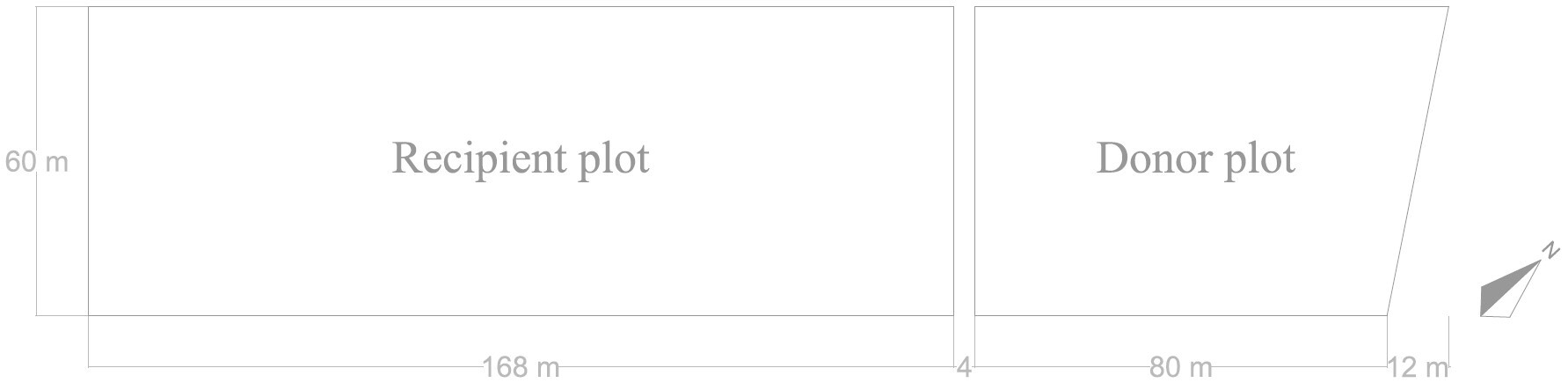


**Fig. 1S** Schematic design of Trial 1. GM donor plot separated from the conventional recipient plot by just a 4 m wide vegetation-cleared strip.


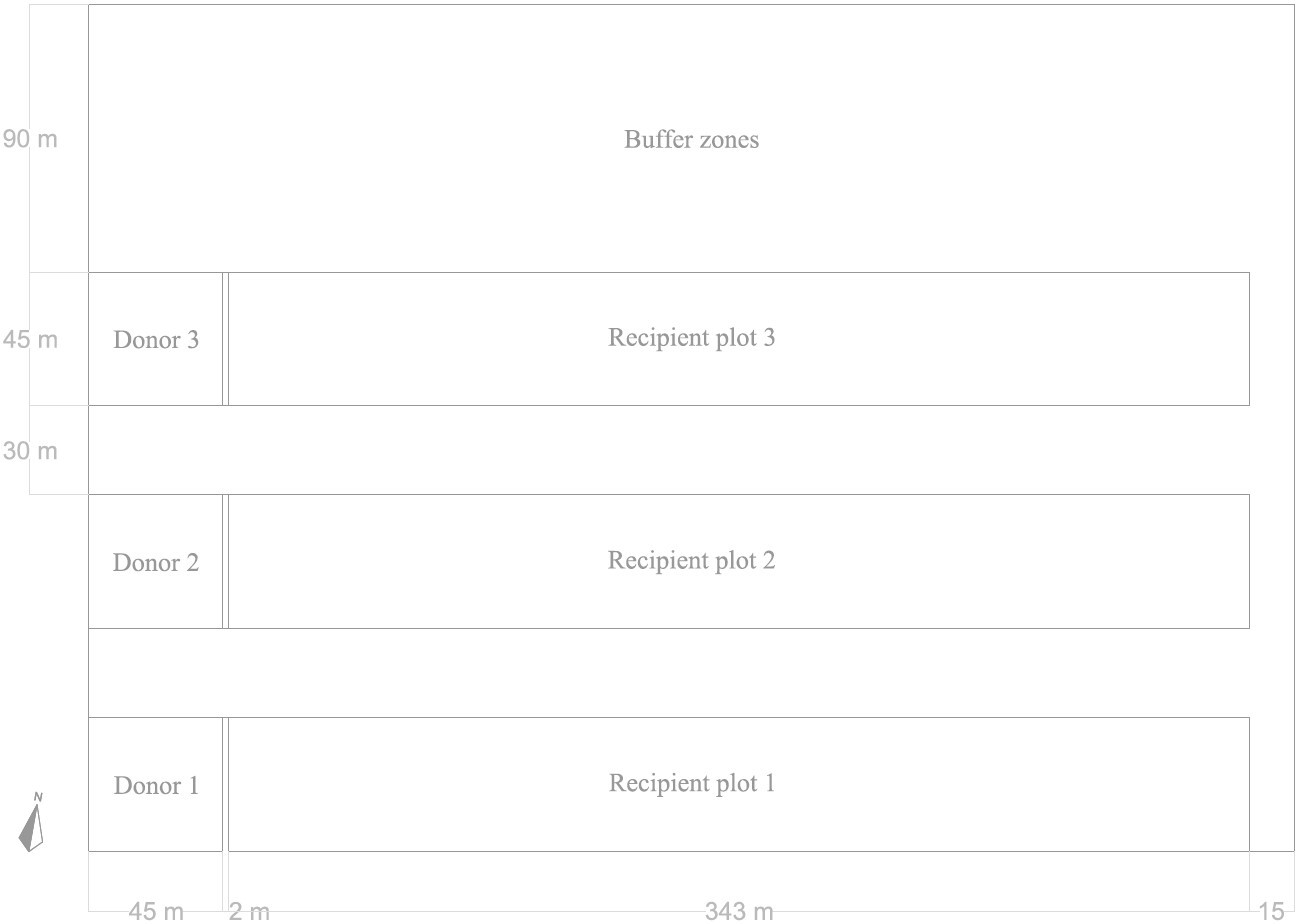


**Fig. 2S** Schematic design of Trial 2. GM donor plots separated from the conventional paired recipient plots by just a 2 m wide vegetation-cleared strip.


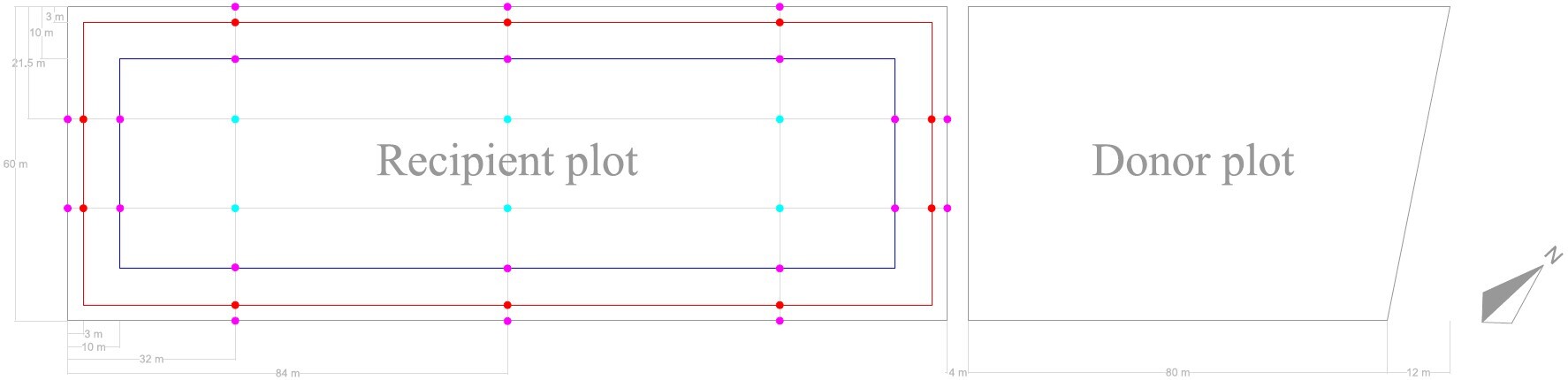


**Fig. 3S** Schematic design of the sampling layout for the recipient plot of Trial 1, based on the methodology of Messeguer et al. (2006).


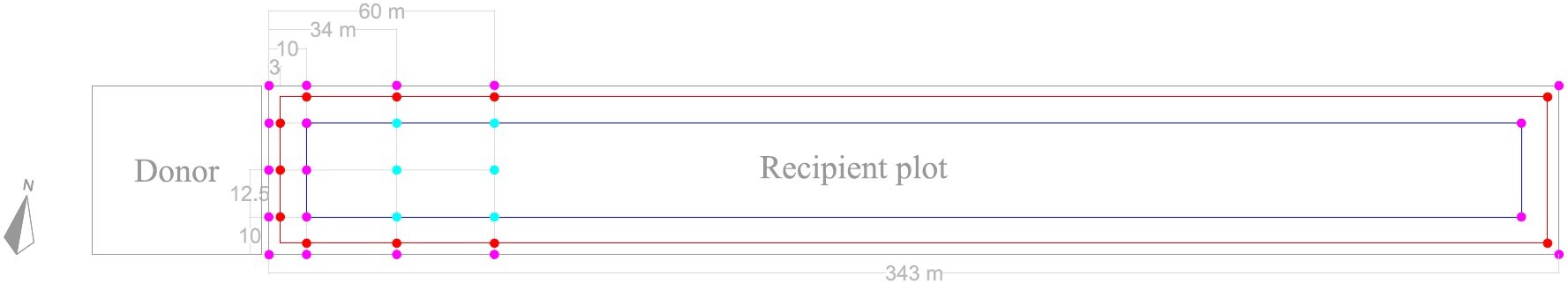


**Fig. 4S** Schematic design of the sampling layout for the recipient plots of Trial 2, based on the methodology of Messeguer et al. (2006)**.**

**Flowering and meteorological data**

The flowering period for Trial 1 was fixed between August 17 and August 23, with its peak on Augst 19. For Trial 2, flowering periods were fixed between July 20 and July 26 for conventional plot 1 (along with the conventional buffer zones and GM donor plots), with its peak on July 22; between July 29 and August 4 for conventional plot 2, with its peak on July 31; and between August 27 and September 2 for plot 3, with its peak on August 29.

In Trial 1, the prevailing wind during the pollen dispersal days and hours had a strong NE component (Fig. 5S) with an average wind speed of 3.57 m/s. A similar pattern was recorded by the AEMET station throughout the month of August (Fig. 9S). In general, during the maximum dispersal hours (between 09:00 a.m. and 02:00 p.m.) by comparison with the pollen dispersal hours (06:00 a.m. to 06:00 p.m.), a trend towards a concentration of wind direction in favour of the N-NE component was found in all summer months (June to September 2013).


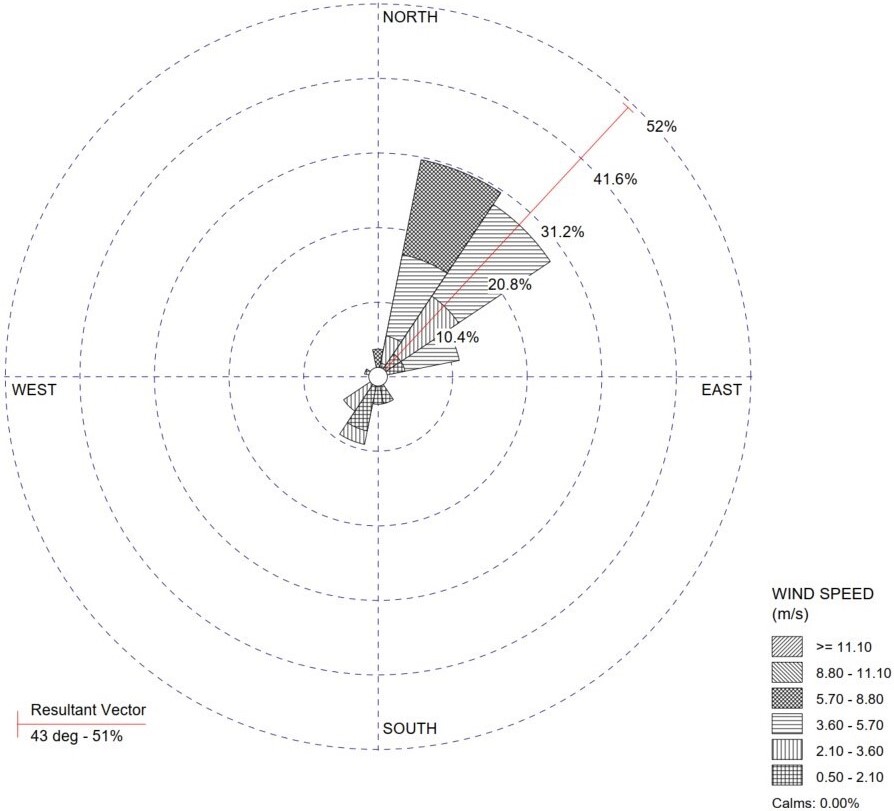


**Fig. 5S** Wind rose diagram for Trial 1. Wind direction and speed (m/s) between 06:00 a.m. and 06:00 p.m. during the flowering period of GM donor and conventional recipient plots of Trial 1 (August 20-23, 2013). Diagram created from the records of the AEMET station SA POBLA SA CANOVA (code B691Y), altitude 40 m, longitude E 3º 1’ 0.1’’, latitude N 39º 44’ 57’’, with the software WRPLOT View Freeware V.8.0.2 (Lakes Environmental Software, Ontario, Canada), 16 wind cardinal directions shown

In Trial 2, the prevailing wind during the flowering period and the pollen dispersal hours (06:00 a.m. to 06:00 p.m.) of conventional recipient plot 1, the buffer zones and the GM donor plots, had a strong SW component (Fig. 6S), with an average wind speed of 4.90 m/s. Such a wind direction pattern was already lessened during the homologous period and time for conventional recipient plot 2 (Fig. 7S), with an average wind speed of 5.06 m/s; and was even weaker during the homologous period and time for conventional recipient plot 3 (Fig. 8S), in this case with an average wind speed of 4.02 m/s. An increasing trend towards the compensation of the prevailing SE component by winds with an almost opposite direction was found, the more pronounced the later in summer. However, a general trend towards a concentration of wind direction in favour of the SW component was also seen during the maximum dispersal hours (between 09:00 a.m. and 02:00 p.m.) by comparison with the pollen dispersal hours (06:00 a.m. to 06:00 p.m.), for all summer months but September in which the trend is rather the opposite (i.e., slight strengthening of the NE component).


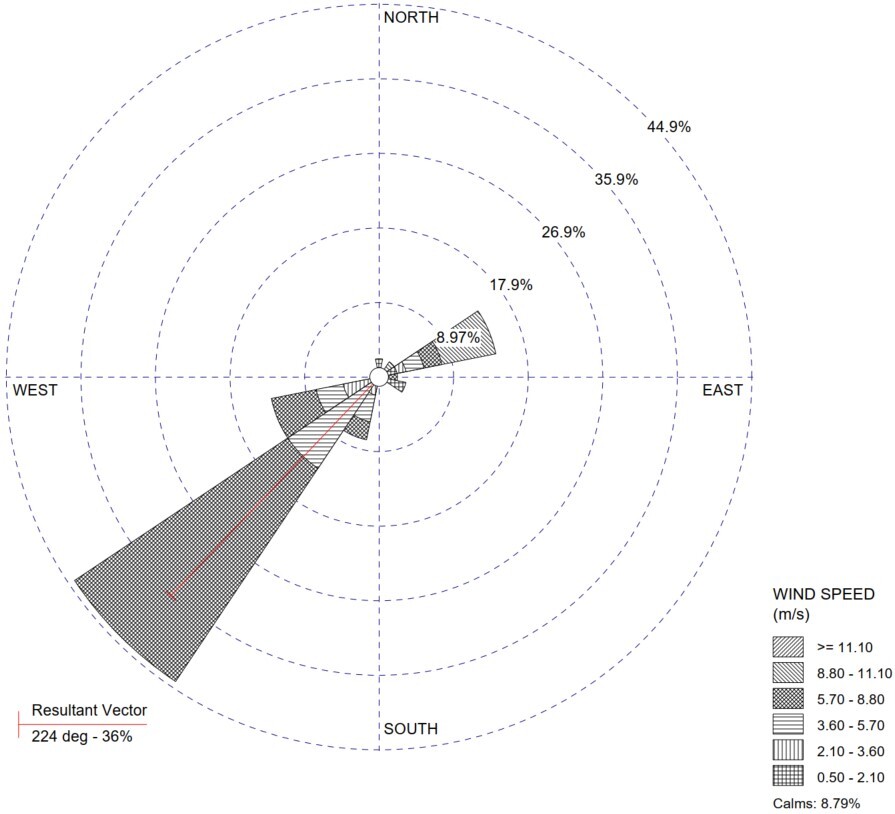


**Fig. 6S** Wind rose diagram for Trial 1. Wind direction and speed (m/s) between 06:00 a.m. and 06:00 p.m. during the flowering period of conventional recipient plot 1 of Trial 2 (July 20-26, 2015). Diagram created from the records of the AEMET station PALMA DE MALLORCA/SON SAN JUAN (code B278), altitude 8 m, longitude E 2º 44’ 12,1’’, latitude N 39º 33’ 39’’, with the software WRPLOT View Freeware V.8.0.2 (Lakes Environmental Software, Ontario, Canada), 16 wind cardinal directions shown


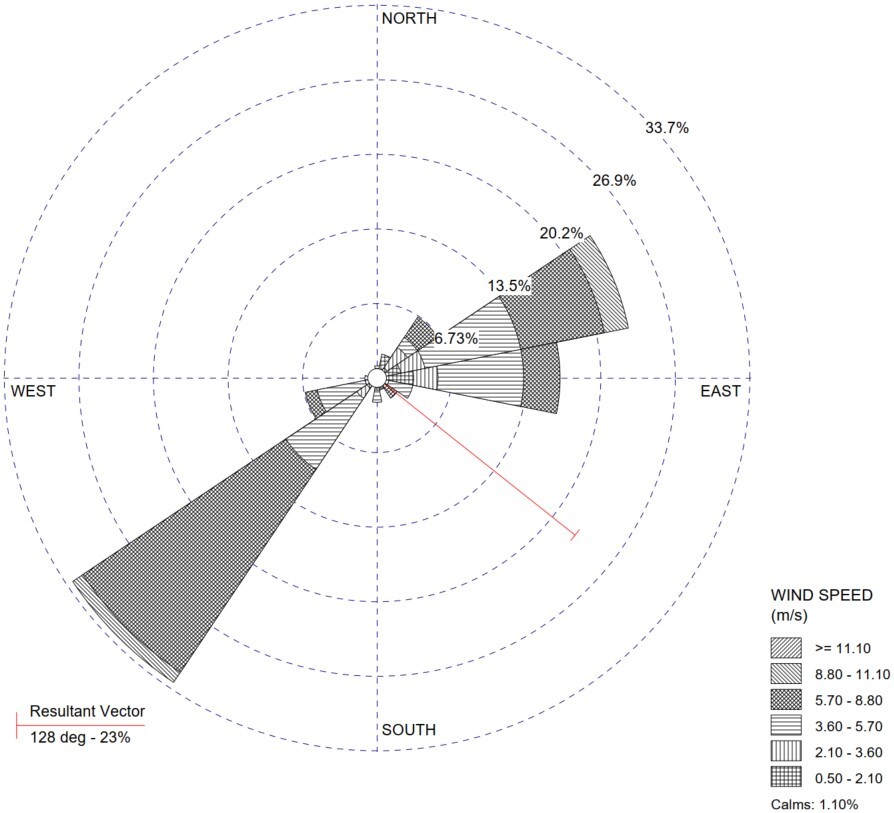


**Fig. 7S** Wind rose diagram for Trial 1. Wind direction and speed (m/s) between 06:00 a.m. and 06:00 p.m. during the flowering period of conventional recipient plot 2 of Trial 2 (July 29 to August 4, 2015). Diagram created from the records of the AEMET station PALMA DE MALLORCA/SON SAN JUAN (code B278), altitude 8 m, longitude E 2º 44’ 12,1’’, latitude N 39º 33’ 39’’, with the software WRPLOT View Freeware V.8.0.2 (Lakes Environmental Software, Ontario, Canada), 16 wind cardinal directions shown


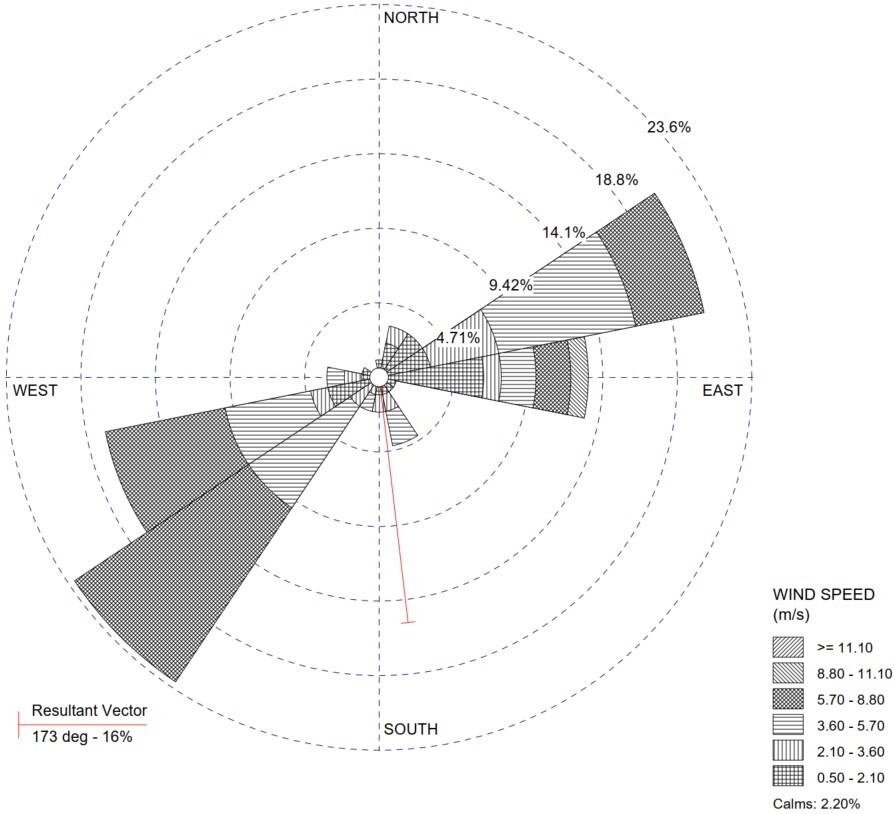


**Fig. 8S** Wind rose diagram for Trial 1. Wind direction and speed (m/s) between 06:00 a.m. and 06:00 p.m. during the flowering period of conventional recipient plot 3 of Trial 2 (August 27 to September 2, 2015). Diagram created from the records of the AEMET station PALMA DE MALLORCA/SON SAN JUAN (code B278), altitude 8 m, longitude E 2º 44’ 12,1’’, latitude N 39º 33’ 39’’, with the software WRPLOT View Freeware V.8.0.2 (Lakes Environmental Software, Ontario, Canada), 16 wind cardinal directions shown


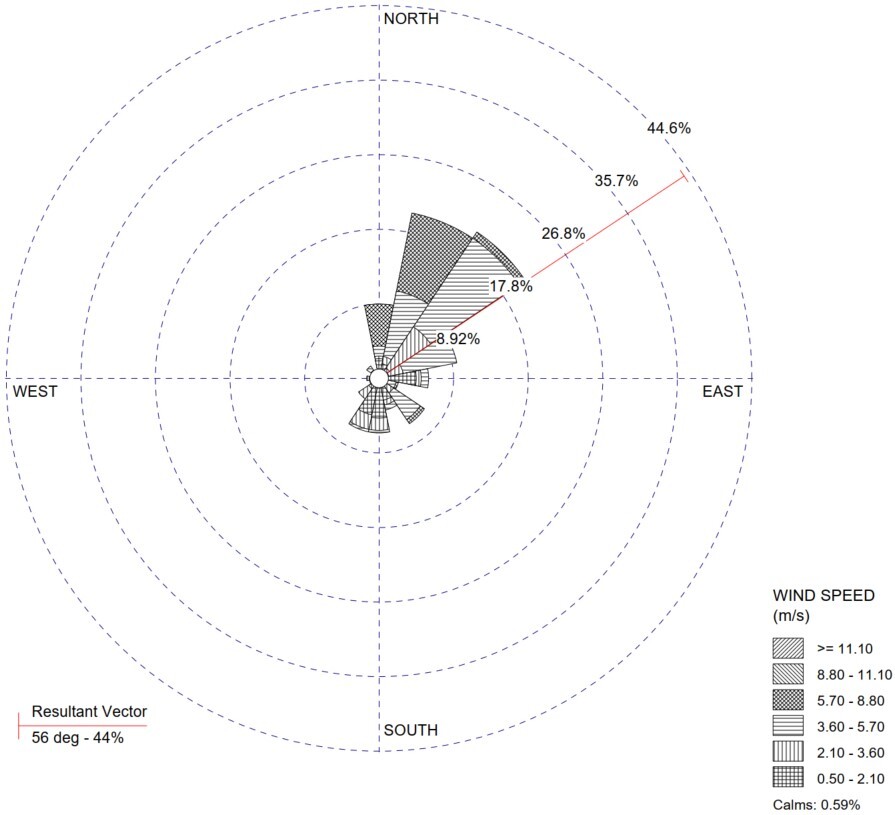


**Fig. 9S** Wind rose diagram for Trial 1. Wind direction and speed (m/s) in August 2013 between 06:00 a.m. and 06:00 p.m. Diagram created from the records of the AEMET station SA POBLA SA CANOVA (code B691Y), altitude 40 m, longitude E 3º 1’ 0.1’’, latitude N 39º 44’ 57’’, with the software WRPLOT View Freeware V.8.0.2 (Lakes Environmental Software, Ontario, Canada), 16 wind cardinal directions shown

Rose diagrams portraying wind speed and direction during the flowering period and the pollen dispersal hours for July, August and September are shown in Figs. 10S, 11S, and 12S.


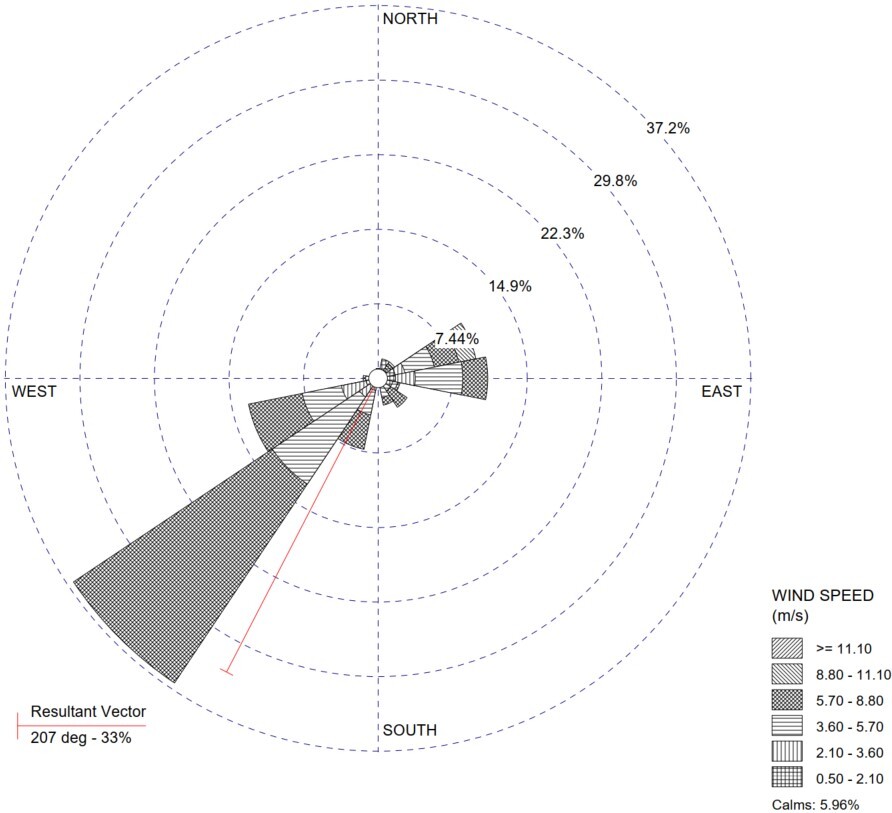


**Fig. 10S** Wind rose diagram for Trial 2. Wind direction and speed (m/s) in July 2015 between 06:00 a.m. and 06:00 p.m. Diagram created from the records of the AEMET station PALMA DE MALLORCA/SON SAN JUAN (code B278), altitude 8 m, longitude E 2º 44’ 12,1’’, latitude N 39º 33’ 39’’, with the software WRPLOT View Freeware V.8.0.2 (Lakes Environmental Software, Ontario, Canada), 16 wind cardinal directions shown


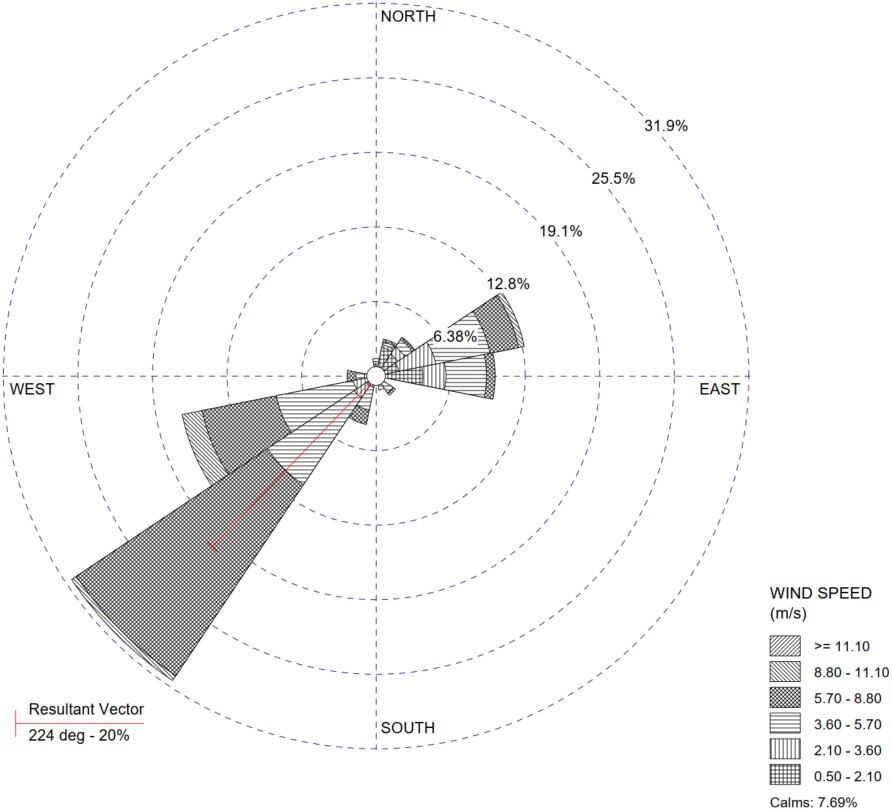


**Fig. 11S** Wind rose diagram for Trial 2. Wind direction and speed (m/s) in August 2015 between 06:00 a.m. and 06:00 p.m. Diagram created from the records of the AEMET station PALMA DE MALLORCA/SON SAN JUAN (code B278), altitude 8 m, longitude E 2º 44’ 12,1’’, latitude N 39º 33’ 39’’, with the software WRPLOT View Freeware V.8.0.2 (Lakes Environmental Software, Ontario, Canada), 16 wind cardinal directions shown


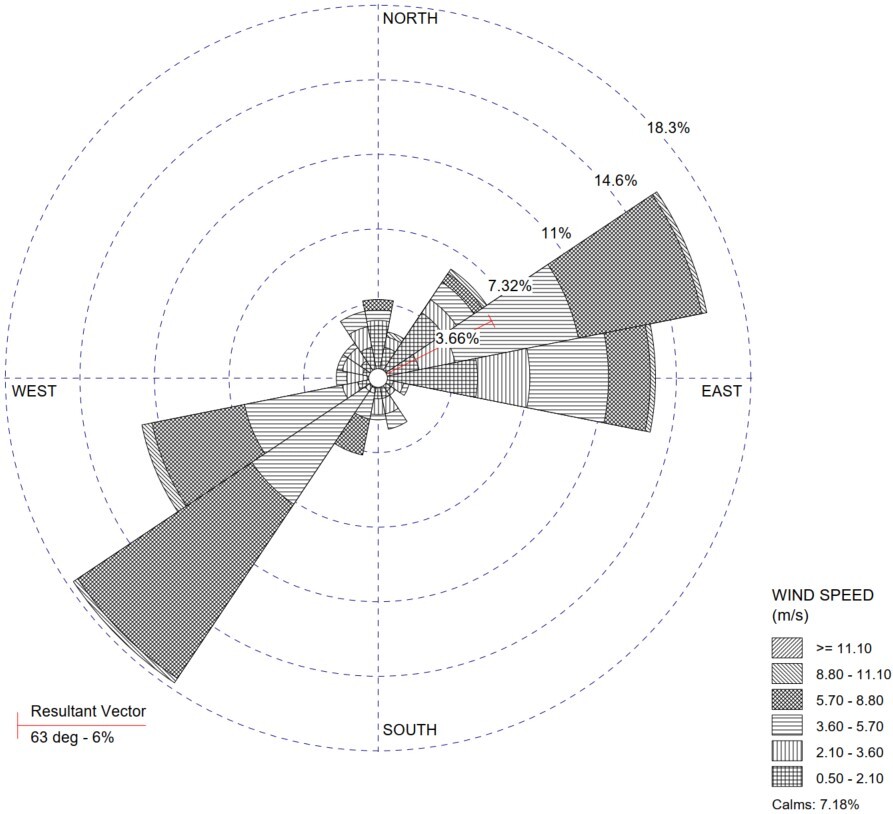


**Fig. 12S** Wind rose diagram for Trial 2. Wind direction and speed (m/s) in September 2015 between 06:00 a.m. and 06:00 p.m. Diagram created from the records of the AEMET station PALMA DE MALLORCA/SON SAN JUAN (code B278), altitude 8 m, longitude E 2º 44’ 12,1’’, latitude N 39º 33’ 39’’, with the software WRPLOT View Freeware V.8.0.2 (Lakes Environmental Software, Ontario, Canada), 16 wind cardinal directions shown

**Table 1S** GM distribution across the sampling points of recipient plot of Trial 1 according to the sampling design shown in Fig. 3S. Each row portrays both the distance (m) from the border (of the recipient plot) adjacent to the donor (GM) plot, and the GM content (%) measured on each of the samples

| **Distance (m)** | **%MON810** |
| --- | --- |
| 0 | 21.75 |
| 0 | 16.78 |
| 3 | 10.40 |
| 3 | 8.29 |
| 10 | 6.36 |
| 10 | 5.87 |
| 32 | 0.67 |
| 32 | 0.19 |
| 32 | 0.48 |
| 32 | 3.98 |
| 32 | 2.47 |
| 32 | 0.00 |
| 32 | 0.00 |
| 32 | 0.00 |
| 84 | 0.40 |
| 84 | 0.17 |
| 84 | 0.22 |
| 84 | 0.10 |
| 84 | 0.00 |
| 84 | 0.00 |
| 84 | 0.00 |
| 84 | 0.00 |
| 136 | 0.52 |
| 136 | 0.00 |
| 136 | 0.00 |
| 136 | 0.00 |
| 136 | 0.00 |
| 136 | 0.00 |
| 136 | 0.00 |
| 136 | 0.00 |
| 158 | 0.00 |
| 158 | 0.04 |
| 165 | 0.00 |
| 165 | 0.00 |
| 168 | 0.00 |
| 168 | 0.00 |

**Table 2S** GM distribution across the sampling points of recipient plots of Trial 2 according to the sampling design shown in Fig. 4S. Each row portrays both the distance (m) from the borders (of the recipient plots) adjacent to the respective donor (GM) plots, and the GM content (%) measured on each of the samples for recipients 1, 2 and 3

| **Distance (m)** | | **%MON810** | | |
| --- | --- | --- | --- | --- |
|  |  | **Recipient 1** | **Recipient 2** | **Recipient 3** |
| 0 | 3.46 | | 2.74 | 0.00 |
| 0 | 39.69 | | 2.01 | 0.00 |
| 0 | 5.68 | | 6.18 | 0.00 |
| 0 | 4.92 | | 0.21 | 0.00 |
| 0 | 46.80 | | 31.66 | 0.00 |
| 3 | 0.16 | | 0.69 | 0.00 |
| 3 | 2.94 | | 0.38 | 0.00 |
| 3 | 1.78 | | 0.36 | 0.03 |
| 10 | 0.13 | | 0.19 | 0.00 |
| 10 | 1.22 | | 0.25 | 0.05 |
| 10 | 2.74 | | 0.00 | 0.00 |
| 10 | 0.51 | | 0.17 | 0.00 |
| 10 | 0.33 | | 3.49 | 0.00 |
| 10 | 0.24 | | 0.17 | 0.08 |
| 10 | 0.19 | | 24.84 | 0.00 |
| 34 | 0.00 | | 0.00 | 0.00 |
| 34 | 0.00 | | 0.00 | 0.00 |
| 34 | 0.00 | | 0.00 | 0.00 |
| 34 | 0.00 | | 0.00 | 0.00 |
| 34 | 0.00 | | 0.00 | 0.00 |
| 34 | 0.00 | | 0.00 | 0.00 |
| 34 | 0.00 | | 0.00 | 0.00 |
| 60 | 0.00 | | 0.00 | 0.00 |
| 60 | 0.00 | | 0.00 | 0.00 |
| 60 | 0.00 | | 0.00 | 0.00 |
| 60 | 0.00 | | 0.00 | 0.00 |
| 60 | 0.00 | | 0.00 | 0.00 |
| 60 | 0.00 | | 0.00 | 0.00 |
| 60 | 0.00 | | 0.00 | 0.00 |
| 331 | 0.00 | | 0.00 | 0.00 |
| 331 | 0.00 | | 0.00 | 0.00 |
| 338 | 0.00 | | 0.00 | 0.00 |
| 338 | 0.00 | | 0.00 | 0.00 |
| 341 | 0.00 | | 0.00 | 0.00 |
| 341 | 0.00 | | 0.00 | 0.00 |
